# Developing a protocol for determining the competitive advantage of clinical specialty and sub-specialty mission differentiation in Iran 

**DOI:** 10.30476/jamp.2020.86151.1221

**Published:** 2020-10

**Authors:** SARA SHAFIAN, PEIGHAM HEIDARPOOR, MARYAM OKHOVATY, SHAHRAM YAZDANI

**Affiliations:** 1 Virtual School of Medical Education and Management, Shahid Beheshti University of Medical Sciences, Tehran, Iran; 2 Neuroscience Research Center, Institute of Neuropharmacology, Kerman University of Medical Sciences, Kerman, Iran

**Keywords:** Clinical medicine, Hospital departments, Qualitative research, Academies and institutes

## Abstract

**Introduction:**

The differentiation as a process occurs when new functions emerge in a system, and for this reason the university system is bound to diversify. With the advancement of knowledge and increase in competition, it has become a challenging issue and an inevitable necessity. This study aimed at identifying the dimensions of the differentiation of Iran's medical sciences universities through determining a protocol of the competitive advantage and scientific strength of clinical specialty and sub-spatiality departments of research activities.

**Methods:**

This was a mixed-methods study; the qualitative study was carried out using a conventional content analysis method. We held a focus group discussion to develop a protocol of scientific strength and competitive advantage for mission differentiation. We used purposive sampling, in February 2019. The sessions were audio-taped. We analyzed the data by considering the verbatim transcribed document of the audio recorded discussions using conventional content analysis method for theme development. In the second stage, we implemented the proposed protocol in the first stage through the Scientometrics System for all 66 colleges and universities of the Ministry of Health and Medical Education.

**Results:**

The results of the first stage includes a protocol, 4 indices and two formulae for scientific strength and competitive advantage proposed by the expert participants and an executive model designed to clinical specialty and sub-specialty of the college and universities of medical sciences in the research activities. In the second stage, the scientific strength and competitive advantage was calculated for all universities of medical sciences. The results indicated that some universities, for instance, Tehran, Shahid-Beheshti, Iran, Shiraz, Isfahan, etc. had the most competitive advantages among the academic clinical specialty and sub-specialty departments.

**Conclusion:**

Besides teaching and research, universities should contribute to local socio-economic development, in the growing conviction that scientific research results and educational skills are crucial for the economic growth of nations. The enhancement of high-quality education and excellence in teaching will be consistent and sustained in research-intensive universities. It should strongly promote the integration and relatedness of teaching and research as an essential characteristic of the university.

## Introduction

Universities are institutions that do important functions in all societies; teaching and research are clearly two fundamental and dominant missions of universities. The growing demand for higher education after the end of World War II and its effects on expanding the capacity of higher education institutions across the world has led to a shift in the nature of the university from elite to mass higher education institutions ( 1 ). Indeed, most countries need higher education institutions and programs that are responsive to the diverse needs of their community, that is different functions and missions should be considered for higher education institutions ( 2 ). The dimensions of teaching and learning, research involvement and knowledge exchange reflect the core functions of higher education institutions ( 3 ). 

The relationship between education and research especially in postgraduate programs has recently been highlighted so that a new term- ‘research-teaching nexus’- has been coined. In other words, teaching itself is just one element ( 4 ). Some methods such as problem-based learning and evidence-based practice can prove that education without research is meaningless. Elken and Wollscheid (2016) conducted a review of the literature on the relationship between research and education, referring to the range of typologies and indicators ( 5 ). Fung and Gordon (2016) found that leading research-intensive universities in the UK were increasingly rewarding education-focused leaders with promotions ( 6 ). Becker and Kennedy (2005) believe that although most research has addressed the benefit of a research-rich culture to students, the researchers benefit from teaching, as well ( 7 ). 

The differentiation can be considered a process in which new entities emerge in a higher education system resulting in more system diversity ( 8 , 9 ), or a process whereby a social unit changes to two or more units. According to this view, new social units are structurally distinct from each other, but their performance is equivalent to the original unit ( 10 ). The concept of differentiation in higher education has been widely discussed ( 11 - 16 ). There is a wide range of examples of innovative practices and specialized programs in mission differentiation around the world. For example, the University of Waterloo is a world leader in the field of cooperative education and McMaster has been a leader in problem-based education in its medical programs ( 17 ). 

Competition among higher education institutions is sometimes identified as a stimulus for differentiation ( 18 ). Competitive advantage in higher education came from the United States as higher education moved from elite to mass, a system including a diverse range of community college institutions and prominent research universities ( 18 , 19 ). The highlight of competition in the American higher education system has actually been to encourage universities to compete through differentiation. In fact, this research is a start for medical sciences universities to compete on a clean spot ( 20 ). There are various models in the world for calculating the competitive advantage and the point of distinction of higher education institutions such as the Porter model (2011) ( 21 ), the Zwanziger model (1996) ( 22 ), the Herfindal index (1950) ( 23 ), the Balassa, Specialized index (1965) ( 24 ), which are indicators that measure the point of differentiation in higher education. These indicators and models measure differentiation in higher education institutions in the dimension of education, and none is applicable to research.

 The evaluation of scientific products of universities, groups and organizations is not a new subject, and with the advancement of knowledge and increasing competition in this field, it becomes a challenging issue and an undeniable necessity. One of the most common ways to evaluate scientific products is to use methods of scientometrics related to the production and dissemination of knowledge and technology. Scientometrics is the science of measuring science using quantitative methods and models ( 25 ), and helps to analyze quantitative aspects of the scientific production and use of the information for better understanding of scientific research ( 26 ). 

In Iran, in 1849, the first modern course of Medicine at Dar-ol-Fonoon School was founded, and the pioneering graduates started the practice of modern medicine in 1856. In 1918, Dar-ol-Fonoon was renamed to College of Medicine, and in 1934 it turned into the School of Medicine of the University of Tehran. At present, higher education systems for medical sciences have amounted to 66 public colleges and universities of medical sciences. All these institutions are under the supervision of the Ministry of Health and Medical Education (MOHME) ( 27 , 28 ). 

A diverse range of higher education institutions with different missions allows the over-all system to meet students’ needs, provide opportunities for social mobility, meet the needs of different labor markets, serve the political needs of the interest groups, increase level of higher education institutions effectiveness, and offer opportunities for experimenting with innovation ( 29 ). However, in recent years, MOHME attempted to have mission-orientated universities. In this regard, the "packages of transformation and innovation in medical sciences" have been developed and implemented with specific missions to the 10 regions of high education spatial planning program, which were not based on the study of the capacities and empowerment of college, universities and regions ( 30 ). The lack of diversity or de-differentiation occurs because of the policy and professional factors which contribute to increasing convergence or homogeneity within the higher education system leading to “academic” or “mission” drift ( 16 ). In this regard, Universities, and particularly the top research-intensive universities, are the higher education institutions that relate most directly to the global knowledge economy. It is these research-intensive universities that might qualify as “world class” institutions in their respective countries and most likely to be recognized in the international rankings ( 31 ).

Therefore, due to the importance and necessity of mission differentiation and no previous studies with a generalized and agreed framework for differentiation and diversity in medical sciences universities in Iran, we conducted this study to identify the dimensions of the differentiation of Iran's medical sciences universities through scientific strength and competitive advantages of research activities in clinical specialty and sub-spatiality departments. The findings of this study can help the MOHME policy-makers to make appropriate planning for the allocation of missions in the field of research and in order to get an appropriate place at national and international levels.

## Methods

We used a mixed methods design ( 32 - 33 ) that incorporated both qualitative (focus group discussion) techniques and quantitative (scientometric) study, ( 34 - 37 ). A protocol of scientific strength and competitive advantage in focus groups session was determined and was implemented during the scientometric study. The data were collected in February-April 2019. 

This study was conducted in Iran within the context of postgraduate training programs of the academic clinical specialties and sub-specialties departments in 66 colleges and universities of medical sciences. In this regard, we explored and identified opinions of research participants by involving the faculty members of Shahid-Beheshti University of Medical Science through focus group discussion (FGD) and used research activity data of the faculty members of the colleges and universities of medical sciences, using scientometric systems MOHME. 

In first stage, FGD was used to develop a protocol for determining the scientific strength and competitive of clinical specialty and sub-spatiality departments. Literature review showed that the focus group method of investigation is used as an explanatory data collection technique ( 32 ). The participants were selected using purposive sampling. In this technique, the participants are selected on the premise of a purpose in the mind of the researcher and the sample is then selected to encompass the interested participants and excludes those who do not suit the purpose ( 33 ). 

At the beginning of the meeting, the research objectives were introduced and the general information about the research and the meeting time were also explained. In addition, a full description of the meeting audio-recording and everyone’s obligation to contribute was provided and the participants were ensured that the information remained confidential and anonymous. In this regard, during the meeting, one of the researchers conducted discussions keeping neutrality and lack of judging, encouraging the contributors to discuss the topic, interact with each other and express their opinions. The other researcher served as an observer, recording the interactions between individuals. After the focus group meeting, the audio-recording was implemented by one of the researchers. The validity, transferability, reliability and verifiability criteria were considered in the study. For validity, the researchers participated in all stages of the study. In addition, the process of data collection and analysis was performed shortly after the FGD. In relation to the transferability criterion, it was attempted to provide a comprehensive description of the study. Regarding reliability, data analysis was performed by two researchers and reviewed by a third person. An external observer was used to examine the data analysis in order to ensure the criterion of verification. We classified each statement in a matrix and searched for themes that summarized the various statements. The consistency of the matrix was checked by the researchers by coding the transcripts again while looking for blanks or inconsistencies that did not fit in the themes and establishing whether the themes were exclusive. Contradictory statements were also explicitly searched for and consensus was reached through discussion. No theoretical framework was used during the coding procedure. 

 In the second stage, we implemented the protocol proposed in the first stage. We extracted the research activity data related to faculty members of clinical specialty and sub-spatiality departments from Scientometrics System MOHME including demographic characteristics, college and university name, H index, total of citations, ranking at national level in fields and specialized fields names. Indicators and formulae were modeled in Excel software. In order to examine the protocol, a pilot calculation was done on the data by Shahid Beheshti University of Medical Sciences, which proved the accuracy. Calculations done for all faculty members of 66 college and universities of medical sciences are available in the MOHME Scientometric System ( 34 ). At this stage, the accuracy of data was examined; the data by the research team meetings and the data transfer were complete. After analyzing the scientometric data of all college and universities, the results were extracted based on the scientific strength Index and the competitive advantage index of results. The Scientometric data were analyzed using Microsoft Excel 2019 and Tableau software version 2018.3. Descriptive statistics (frequencies and percentages) were provided.

## Results

Five faculty members took part in the focus groups from Shahid-Beheshti University of Medical Sciences. Five research participants took part in the research; according to the literature, the number of research participants in a FGD meeting depends on the issue, the expected phenomenon characteristics and data saturation ( 38 , 39 ), and an ideal group size in FGD ranges from four to eight people ( 35 ). The mean age of the participants was 43.1 and three participants were female and the session lasted for approximately 120 minutes. In terms of academic rank, 1 was an assistant professor, 2 were associate professors and 2 were full professors. In order to gather rich data, the participants were selected 1) with specialties related to medical education, medical information and clinical specialty and sub-spatiality, 2) with a background in scientometrics and 3) willingness to participate in the study. It should be noted that because of the large number of departments and lack of science strength and competitive advantage in some college and universities only the colleges and universities with an “inclusive advantage” and “competitive advantage” were included, based on table (1). 

 The data obtained from the FGD analysis were grouped into a protocol including 2 formulae (SSI & CAI) and 4 indices (T_10_C, T_10_C/N, T_10_CU/N, H_2_ Index) and three main categories related to executive model ( Table 1).

**Table 1:** Executive model of scientific strength and competitive advantage in research activities based on the focus group discussions.

Main Categories	Sub-categories
Model input	Using scientific indices of faculty members as the main arm of medical sciences universities in research.
	Using academic membership website as the main reference for extracting research data from universities as the only valid and existing system in this field.
	Calculations should only be considered for medical sciences field and non-medical sciences calculations were excluded.
	Performing calculations for medical institutions including colleges and universities of medical sciences and excluding other institutions in the science system.
Model implementation process	Using the Scopus set in the calculations and not including the Google scholar set.
	The computation is based on the fields contained in the field of the "academic discipline" and the differentiation of the academic disciplines recorded in the system.
	The rankings of faculty members in the scientometric website were considered to be based on the h-index.
	Fields in computing were expected to have more faculty members than one university.
Model output	Competitive power over 5 was considered an "exclusive advantage" of the universities in the medical sciences.
	Competitive power was defined above 0.3 to 5 as a "competitive advantage" of the universities in medical sciences.
	Considering competitiveness between 0.1 and 0.3 as the "mild competitive advantage" of medical universities.
	Considering the lower competitiveness of 0.1 as a "lack of competitive advantage" for medical universities.

**T_10_C:** Total university team citations; In order to calculate this index, the individuals in the group were sorted by H Index from high to low; the index was calculated from the sum of the top 10 citations of the group.

**T_10_C/N:** Ratio of citations of the university team to the national team; to calculate this index, the sum of citations of the top 10 academic staff in the university was divided by the sum of citations of the top 10 academic staff in the national.

**T_10_CU/N:** University contribution from national team citations; To calculate this index, the total number of citations of the academic staff from the top 10 national in the university is divided by the sum of the top 10 citations in the national. This index was designed to control calculations for each field; the sum of value all college and universities was equal to 1. The value <1>, showed an error in the calculations.

**H_2_ Index:** One of the recent and strong indices is h-index. In 2005, Hirsch suggested h-index as a simple and useful way to describe the scientific output of researchers (28). The number of articles published and the number of citations are both considered in this index. Brown et al. (2004) proposed an index of the type of Hirsch as index “h2”, which shows the largest number of individuals of a group (N) with the H index larger or equal to N (29).

 Therefore, for finalizing the protocol 4 indices were included in the formulae Scientific Strength Index (SSI) and Competitive Advantage Index (CAI) and the calculations were done for all faculty members of clinical specialty and sub-specialty based on these two formulae: 

$\mathrm{Scientific Strength Index(SSI)}=\frac{\mathrm{T10C}}{\mathrm{T10N}}\times \mathrm{h2}$

$\mathrm{Competitive Advantage Index(CAI)}=\frac{\mathrm{SSI C/UMS}}{\mathrm{SSIRival}}$

*UMS: College and University Medical Science*

*SSI Rival: the highest SSI (first rank) in each field divide to others and the second rank divide into the first rank.*

**Figure 1:**
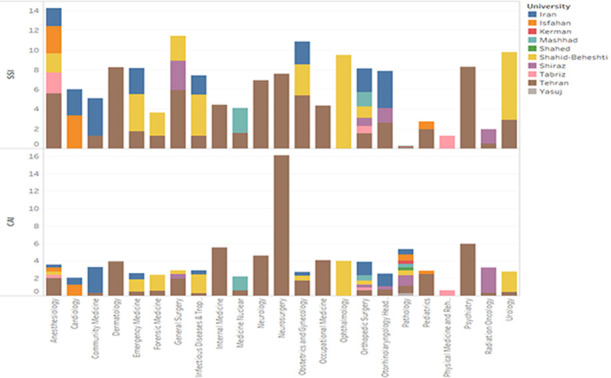
Scientific Strength Index (SSI) and competitive advantage index (CAI) and type of competitive advantage of Iranian clinical specialty department.

As the results show, among the academic clinical specialty departments, Tehran (in 20 specialties), Shahid-Beheshti (in 10 specialties), Iran (in 10 specialties), Shiraz (in 5 specialties), Isfahan (in 4 specialties), Mashhad (in 3 specialties), Tabriz (in 3 specialties), Kerman (in 1 specialty), had the most academic departments for competitive advantages. Also, some small universities such as Shahed (in 1 specialty), Yasuj (in 1 specialty) had also competitive advantages in some academic departments that had to be considered. There were more competitive advantages in the clinical sub-specialty departments of universities. For example, Tehran (in 14 sub-specialties), Shahid-Beheshti (in 13 sub-specialties), Shiraz (in 7 sub-specialties), Isfahan (in 5 sub-specialties), Iran (in 4 sub-specialties), Mashhad (in 3 sub-specialties), Tabriz (in 2 sub-specialties), and smaller universities such as Babol, Gilan, Mazandaran had the competitive advantage in 1 sub-specialty. The lack of awareness about the advantages of these universities is the point that should be considered.

**Figure 2:**
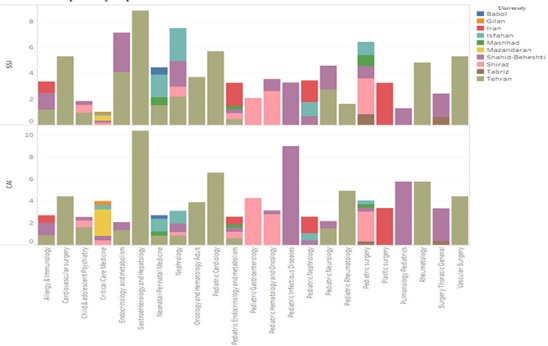
Scientific Strength Index (SSI) and competitive advantage index (CAI) and type of competitive advantage of Iranian clinical sub-specialty department

According to Figures 1 and 2, in some sub-specialties only a few universities have the competitive advantage, e.g. in dermatology, internal medicine, neurosurgery occupational medicine, neurology, psychiatry only Tehran University of Medical Sciences had the competitive advantage and Shahid-Beheshti was the only university having the competitive advantage for Ophthalmology and Tabriz University of Medical Sciences had the competitive advantage for physical medicine. As this chart shows, the specialties such as pathology, orthopedic surgery and anesthesiology had competitive advantages in more universities. It means that all ten universities had the competitive advantage in pathology.

**Figure 3:**
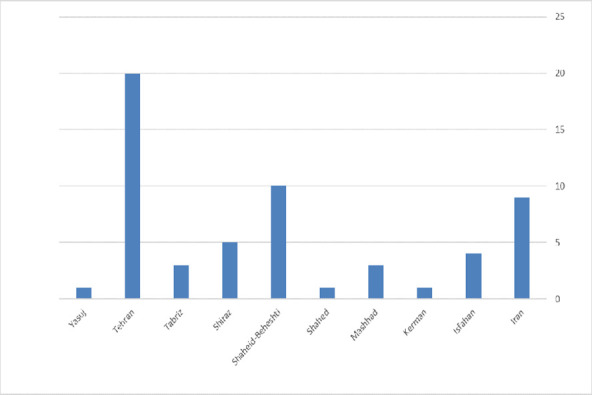
The number of clinical specialty departments having a competitive advantage according to institution

Figure 3 shows the universities having competitive advantage in the specialties: Tehran (in 20 specialties), Shahid Beheshti (in 10 specialties) and Iran (in 8 specialties). Kerman, Yasuj and Shahed universities had the competitive advantage in 1 specialty. These three universities had the competitive advantage in pathology.

**Figure 4:**
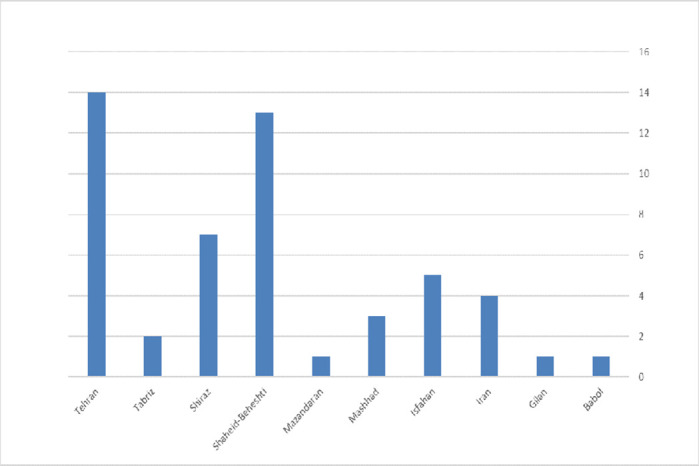
The number of clinical sub-specialty departments having a competitive advantage according to institution

Figure 4 shows the universities having competitive advantage in the sub-specialties: Tehran (in 14 sub-specialties), Shahid-Beheshti (in 13 sub-specialties), Shiraz (in 7sub-specialties), Isfahan (in 5 sub-specialties), Iran (in 4 sub-specialties), Mashhad (in 3 sub-specialties) and Tabriz (in 2 sub-specialties). Mazandaran, Babol and Gillan universities had the competitive advantage in 1 sub-specialty.

## Discussion

Although research and education are widely considered as the primary processes of modern higher education, the link between research and education in higher education has been subject to an ongoing and controversial debate on the nature of the relationship between its strength and directions ( 36 ). The evidence proves a highly complex and multidimensional picture on the research-education relationship so that Elkin puts forward that due to the ambiguous nature of the link between the research-education, the indicators to measure this relationships on higher education in Norway should be seen as “quasi-indicators” rather than full-scale performance indicators ( 5 ).

Clinical specialty and sub-spatiality departments in medical sciences universities of Iran in research activities have a competitive and exclusive advantage. Although Tehran, Shahid-Beheshti, Iran, and Shiraz universities had competitive advantages in most specialty and sub-specialty departments, other universities had a competitive advantage in some departments that can serve as a potential to advance higher education goals. Tehran University of Medical Sciences had the competitive advantage in 10 specialties; Shahid-Beheshti having competitive advantage in 8 specialties followed Tehran University. Three universities, Kerman, Yasuj and Shahed had the competitive advantage in only one specialty. Among the specialties, pathology is the only specialty in which all 10 universities had the competitive advantage. Orthopedic surgery and anesthesiology were the specialties in which 6 and 5 universities had the competitive advantage, respectively.

Mission differentiation has also been a key issue in the international level, with a major focus on the diversity of institutions in the higher education systems. In this regard, higher education systems like Australia have paid more attention to it. Evidence-Based profiles for Australian universities, the U-Map and U-Multirank projects, were initiated in Europe. Each institution profile contains five dimensions: Teaching and Learning, Student Profile, Research Involvement, Knowledge Exchange, and International Orientation ( 37 ). Differentiation in Ontario’s public postsecondary system, where institutions build on and are accountable for their specific strengths, mandates and missions, identifies clear distinctions between universities in terms of their research and teaching missions. The goal is a system that is more cohesive, more sustainable and of higher quality. The internationally competitive University of Toronto had six research-intensive universities, nine mostly undergraduate universities and four “in-between” institutions ( 38 ). Similar frameworks have been implemented in other countries such as Japan, Italy and Saudi Arabia ( 39 - 41 ). Institutional diversity, or differentiation, is one of the most intensely debated topics of higher education studies. Therefore, like the results of the studies of other countries, it can play an effective role in moving the higher education system of Iran toward mission differentiation and diversity of institutions.

Focusing on offering programs with a competitive advantage is an important strategy for success in a highly competitive market of globalized higher education because the higher education institutions have always had a competitive ecosystem in an attempt to achieve high academic standards, achieve academic excellence, and gain international reputation ( 42 ). Focusing on delivering niche programs with a competitive advantage is a critical strategy for succeeding in the competitive higher education systems. Therefore, to achieve sustainable competitive advantage, resources and capabilities should be integrated into higher education institutions ( 43 ). There are different types of competitive advantages in differentiation ( 43 , 44 ). Higher education institutes have challenges to achieve competitive advantage in both national and international levels. For example, changing government policy, continuous student growth, stakeholder demand for quality, change in leadership, new organizational strategy, and financial sustainability are some of the intrinsic challenging factors ( 43 ). 

Teaching and research are the two traditional core activities of any university, but universities have also a major role in more applied fields such as policymaking and wealth creation. Recent rising demands and the changing higher education landscape in a globalized world are accompanied by attempts to define what new activities or objectives of HEIs were or should be institutionalized and supported. The integration of research and teaching has come to be called the third mission of HEIs. The third mission, i.e. the contribution of education to social progress, calls for universities not only to produce new knowledge but also do so with social and economic perspectives in mind ( 45 ).

 As an imperative policy since the emergence of research and development practices in the post-second world war period, third mission models state that, besides teaching and research, universities should contribute to the local socio-economic development, in the growing conviction that scientific research results and educational skills are crucial for the economic growth of nations. All third mission actions are carried out in the belief that the prerequisite for the socio-economic development can arise from research in higher education ( 46 , 47 ). The third mission helps universities to strengthen the ties of universities with industry and society ( 48 ). Therefore, according to the importance of the universities in socio-economic development and the results of this study in competitive advantage universities for differentiated mission, an important industry in which medical sciences universities can operate well and have a lot of socio-economic advantage for health system is the “Medical Tourism” industry. The number of tourists traveling have grown from 529 million in 1995 to 1,235 million in 2016. International tourist arrivals (overnight visitors) worldwide grew 4% in 2019 to 1.5 trillion, based on data reported by destinations around the world. All regions enjoyed an increase in arrivals; The Middle East (+8% growth), followed by Asia and the Pacific (+5%) ( 49 ). 

Every year, 300,000 foreign tourists travel to Iran for medical treatment, with a total of $ 1.2 billion worth of revenues ( 50 ), and it is predicted that 1,400,000 people in 2025 will be attracted to medical tourism ( 51 ). The popularity of Iranian health practitioners in the region and the qualified medical facilities have been another main reason for Iran to be considered as a medical tourism destination ( 52 , 53 ). 

The governance of medical tourism in its various forms is very diverse and complex with a wide range of influential stakeholders (government and non-governmental, individual and institutional). The importance of the government’s participation in developing medical tourism has strongly been identified in South Korea, Malaysia, Croatia, Hong Kong, Singapore and Thailand ( 54 - 58 ). The governments in the major medical tourism destinations regard the medical tourism trade as an important resource for economic and social development ( 58 ). Proper planning and guiding medical tourists in the right direction can help the medical tourism industry in Iran. The results of this study can provide a very effective help in developing this industry to policy makers of MOHME “Health Tourism Council”, because medical tourists can be directed to different places or provinces of Iran to use high quality clinical services with competitive advantage specialty and sub-specialty departments. 

The pattern of differentiation and diversity at the medical sciences universities are based on improving Iran's higher education system. Assigning differentiated missions to medical sciences universities and doing it based on the competitive advantages of medical universities is an effective solution; although implementation seems to be facing some challenges, including the following: 

- The competitive advantages of medical sciences universities in Iran are more environmental and geographical than human and academic. Because of the governmental nature and centralized medical sciences education, human resource composition in universities is very similar in terms of skill and knowledge, and only the quality of presentation differentiates them, so it seems that defining functional and mission differentiation in medical sciences universities is the structural transformation of university policy and governance. Therefore, consideration of the prerequisites and factors necessary for such differentiation is crucial.

- Regarding the integration system in the education of medical sciences and the functions of universities in the medical sciences, there is considerable similarity in the differentiation and separation of missions across the university level with greater complexity.

- Because of the unstable academic development of competitive advantages, it may be highly dependent on the individual, and sometimes one or two faculty members play an important role in the competitive advantage of a university in a particular area.

Limitation

This study was carried out with the limitation of MOHME scientometric system as the only valid reference in Iran in providing data related to the research activities of the faculty members of the medical universities. Extraction of data from this system was accompanied by problems that were solved by holding regular meetings of the members of the research team and cross checking the data several times.

## Conclusion

Iran needs a mission differentiation of higher education system with more transparent information about the specific profile and performance of individual institutions. This study and other studies like this could be helpful, encouraging governments and institutions to go beyond. Universities make strategic choices for reasons not necessarily related to profit-making activities but for a variety of other reasons, including improving academic reputation.
